# Chemobiosis reveals tardigrade tun formation is dependent on reversible cysteine oxidation

**DOI:** 10.1371/journal.pone.0295062

**Published:** 2024-01-17

**Authors:** Amanda L. Smythers, Kara M. Joseph, Hayden M. O’Dell, Trace A. Clark, Jessica R. Crislip, Brendin B. Flinn, Meredith H. Daughtridge, Evan R. Stair, Saher N. Mubarek, Hailey C. Lewis, Abel A. Salas, Megan E. Hnilica, Derrick R. J. Kolling, Leslie M. Hicks

**Affiliations:** 1 Department of Chemistry, University of North Carolina at Chapel Hill, Chapel Hill, NC, United States of America; 2 Department of Chemistry, Marshall University, Huntington, WV, United States of America; University of Colorado Boulder, UNITED STATES

## Abstract

Tardigrades, commonly known as ‘waterbears’, are eight-legged microscopic invertebrates renowned for their ability to withstand extreme stressors, including high osmotic pressure, freezing temperatures, and complete desiccation. Limb retraction and substantial decreases to their internal water stores results in the tun state, greatly increasing their ability to survive. Emergence from the tun state and/or activity regain follows stress removal, where resumption of life cycle occurs as if stasis never occurred. However, the mechanism(s) through which tardigrades initiate tun formation is yet to be uncovered. Herein, we use chemobiosis to demonstrate that tardigrade tun formation is mediated by reactive oxygen species (ROS). We further reveal that tuns are dependent on reversible cysteine oxidation, and that this reversible cysteine oxidation is facilitated by the release of intracellular reactive oxygen species (ROS). We provide the first empirical evidence of chemobiosis and map the initiation and survival of tardigrades via osmobiosis, chemobiosis, and cryobiosis. *In vivo* electron paramagnetic spectrometry suggests an intracellular release of reactive oxygen species following stress induction; when this release is quenched through the application of exogenous antioxidants, the tardigrades can no longer survive osmotic stress. Together, this work suggests a conserved dependence of reversible cysteine oxidation across distinct tardigrade cryptobioses.

## Introduction

Tardigrades are a phylum of eight-legged microscopic invertebrates renowned for their remarkable ability to survive extreme environmental stressors [1–6]. This survival is rooted in their ability to initiate cryptobiosis, a physiological state wherein metabolism slows to near undetectable conditions, enabling long-term survival despite inhospitable conditions [7]. Although some eukaryotes and bacteria are capable of cryptobiosis, no eukaryotes are able to do so across the entirety of their lifespan, including as eggs, juveniles, and adults, or in response to such a broad range of stressors as tardigrades [8]. Thus tardigrades’ ability to survive desiccation, freezing, oxygen starvation, fluctuating osmotic pressure, and ionizing radiation (via anhydrobiosis, non tun-forming cryobiosis, anoxybiosis, osmobiosis, and irradiation-induced dormancy, respectively) is matched by none [9].

Mechanisms of tardigrade survival are still poorly understood. While several cryptobiotes rely on trehalose synthesis as a protectant during dormancy, many tardigrade species produce low or undetectable levels of the disaccharide [10–14]. Tardigrades also lack highly conserved networks for stress regulation, including those that connect hypoxia, genotoxic stress, and oxidative stress to the conserved master regulator target of rapamycin (TOR) [15]. Instead, tardigrades rely on several organism-specific proteins for stress protection, such as the damage suppressor protein (Dsup) that associates with nuclear DNA to protect from ionizing radiation [15]. Additionally, cytoplasmic-, secreted-, and mitochondrial- abundant heat soluble (CAHS, SAHS, and MAHS) proteins, collectively known as tardigrade disordered proteins (TDPs), possess sequences without conservation in other phyla and are essential for tardigrade survival to desiccation via anhydrobiosis [16]. Further, late embryogenesis abundant (LEA) proteins and heat shock proteins (HSP) are widely distributed throughout tardigrade cells, and many are upregulated during tardigrade anhydrobiosis suggesting an essential role in cryptobiosis [17–20].

The hallmark of most tardigrades undergoing cryptobiosis is their remarkable ability to shift into a shriveled anatomical state known as a tun, which they achieve by contracting their limbs, retract along their medial axis, and in the process decrease their internal water stores [21]. Upon exposure to favorable conditions, tardigrades rapidly distend and return to active metabolism. It has long been understood that tardigrades facilitate these anatomical transitions via active processes, yet the mechanism(s) through which tardigrades recognize environmental fluctuations and signal the transitions to enter and exit tuns remains largely unexplored [22]. Tardigrades rely on mitochondrial activity for desiccation survival; using chemical inhibitors to uncouple the mitochondrial electron transport chain prevents tuns from forming following exposure to anhydrobiotic conditions [23]. Further, the ATP-dependent differential phosphorylation of the AMP‐activated protein kinase (AMPK) regulatory network following anhydrobiosis revealed that protein phosphatase 2A activation is essential to successfully induce tuns via anhydrobiosis [24–26]. Mitochondrial electron transport and AMPK regulation, both of which are highly conserved across taxa, engage in crosstalk with reactive oxygen species (ROS). In fact, mitochondrial ROS generation has been directly linked with AMPK activation in mouse embryonic fibroblasts, which is considered to be conserved across taxa [27–30]. When combined with increased accumulation of antioxidant enzymes and the redox homeostasis mediator glutathione following anhydrobiosis [31, 32], evidence suggests oxidative signaling may play a key regulatory role in initiating tun formation.

Herein, novel evidence supporting reversible oxidation as an essential regulatory signal for cryptobiosis is demonstrated in the model tardigrade species, *Hypsibius exemplaris*. Tardigrades are shown to respond to exogenously applied ROS by forming tuns in a dose-dependent manner that is inhibited when cysteine thiols are irreversibly blocked. Thiol-specific fluorescent labeling enabled imaging via confocal fluorescence microscopy with quantification of reduced thiols accomplished through quantitative fluorescent assays. Rapid exposure to reducing conditions causes tun release and death, indicating that the oxidation and reduction of cysteine thiols is accomplished via highly regulated internal networks. Further, quantitative electron paramagnetic resonance spectrometry (EPR) spectroscopy reveals an intracellular release of ROS following stressor induction, that is fatal when blocked with exogenously applied antioxidants. Inhibition of voltage-dependent anion channel protein 2 generates tuns, suggesting ROS control of this ion transporter is likely implicated in tardigrade stress. Together, these data support reversible oxidative signaling as an indispensable regulatory mechanism for tardigrade survival to adverse environments, with a conserved role across distinct cryptobiosis.

## Materials and methods

### Tardigrade husbandry

Cultures of *H*. *exemplaris* (Sciento; Manchester, UK) were reared in 1- or 2-L Erlenmeyer flasks on stationary-phase *C*. *vulgaris*. Culture medium was changed biweekly using a 40-μm Corning™ Sterile Cell Strainers (Thermo Fisher Scientific, Waltham, MA), which retains the tardigrades while allowing algae and waste to flow through. The filter is inverted and rinsed into the same flask with the addition of fresh deionized water. Tardigrades are fed weekly with *C*. *vulgaris* that was grown photoautotrophically under previously established protocols [23]. Cultures were maintained at room temperature on a 12:12-h light:dark cycle using a 7 W (630 lumens) LED lamp [33].

### Chemobiosis induction and concentration dependence

Tardigrades were transferred in minimal media to 35-mm plastic Petri dishes and dosed with concentrations of 750 μM, 1 mM, 2.5 mM, or 5 mM hydrogen peroxide (Thermo Fischer Scientific, Waltham, MA) for 12 h and tun formation was observed with hourly checks. There were 30 animals used per biological replicate, with 4 biological replicates per peroxide concentration. Tardigrades were manually counted every half hour for tun formation. Following tun formation, animals were transferred to new plates containing culturing media and monitored for recovery. Survival for each stressor was defined as tardigrades exhibiting controlled movement of limbs and/or body post-recovery treatment. Surviving tardigrades were counted after 24 h.

### Osmobiosis induction and concentration dependence

Tardigrades (30 animals per replicate, 4 replicates per concentration/condition) were transferred in minimal media (<100 μL) on 35-mm plastic Petri dishes and dosed with working concentrations of 50, 75, 100, and 150 mM CaCl_2_ (Amresco, Dallas, TX) or 225, 300, 450, or 600 mM sucrose (Millipore, Burlington, MA). Tardigrades were manually counted every half hour for tun formation; counting stopped once all conditions were 100% in tuns (12 h for CaCl_2_ and 6 h for sucrose) for 5 and 1 h, respectively, and tun formation was monitored. Animals were transferred to new plates containing culturing media and monitored for recovery. Survival for each concentration was defined as tardigrades exhibiting controlled movement of limbs and/or body post-recovery treatment. Surviving tardigrades were counted after 24 h.

### Confocal bright field imaging

#### Sample preparation

All tardigrades were imaged on microscope slides with tardigrades placed in 20 μL total volume droplets within 0.12 mm deep SecureSeal^TM^ Imaging Spacers (Thermo Fisher Scientific, Waltham, MA) to prevent crushing of tardigrades. Hydrated (active) tardigrades were dosed with 12.5% (v/v) methanol in water (Thermo Fisher Scientific, Waltham, MA) prior to microscopy in order to stop of movement of the tardigrades and allow for high resolution imaging without tardigrade fatalities. Methanol has been previously reported to successfully anesthetize *Caenorhabditis elegans* in a completely reversible manner and with minimal lethality [34]. In house tests reproducibly observed 100% tardigrade survival when dosed with 12.5% v/v methanol. For tardigrades in tuns, it was essential to keep the tardigrades exposed to the tun inducing conditions (for example, tuns induced in 600 mM sucrose were also imaged in 600 mM sucrose). Since tuns do not move, no methanol was needed. After animals were placed inside a spacer, the slides were sealed with a glass coverslip.

#### Imaging

All images were obtained using a Leica SP5 TCSII (Wetzlar, Germany). 10 individual tardigrades were imaged for each condition (hydrated, all tun-forming stressors, and cryobiotes). Tardigrade sizes were estimated by measuring from the most anterior region to the most posterior region on ImageJ [35].

### Cysteine blocking in chemobiosis

Tardigrades (30 animals per sample, 4 replicates per condition) were collected in microcentrifuge tubes were subjected to a 30-min incubation in 30 μM iodoacetamide (IAM) (Thermo Fisher Scientific, Waltham, MA), 30 μM N-ethylmaleimide (NEM) (Thermo Fisher Scientific, Walthman, MA), or water (control). Both IAM and NEM irreversibly bind to reduced cysteines. The 30 μM concentration was chosen because this exposure did not result in tardigrade fatalities in ideal (non-stressed) conditions after 24 h of monitoring for irreversible cysteine thiol binding. Following cysteine blocking, tardigrades were rinsed twice with 1 mL of deionized water to remove remaining blocking reagent and centrifuged at 10,000 x*g* for 3 min. Following the second rinse, the supernate was removed, and tardigrades were transferred to 35-mm Petri dishes in less than 20 μL final volume. The dish was then filled with 750 μM peroxide in culturing media, and the tardigrades were observed for tun formation after 12 h (chosen as earlier experiments show 100% of tardigrades in tuns after 12 h). Tardigrades exposed to NEM and peroxide were imaged using a Leica SP5 TCSII (Wetzlar, Germany). Tardigrades were added to microscope slides in 20 μL total volume droplets within 0.12 mm deep SecureSeal^TM^ Imaging Spacers (Thermo Fisher Scientific, Waltham, MA). The concentration of peroxide was maintained on the slide in order to prevent premature release from tuns. After animals were placed inside a spacer, the slides were sealed with a glass coverslip.

### Induction of cryobiosis

Tardigrades were transferred in droplets of minimal media (<40 μL) on a 35-mm plastic Petri dish and placed in Thermo Fisher Scientific Nalgene ‘Mr. Frosty’ Freezing Container containing 70% isopropyl alcohol (Thermo Fisher Scientific, Waltham, MA) at -80 ⁰C for 4 h. Mr. Frosty freezing containers limit cooling to -1°C per minute. The tardigrades were left for 4 h as this ensured the temperature would have reached -80 ⁰C. Tardigrades were subsequently removed from the apparatus, thawed at room temp, and monitored for recovery. Repeated trials showed 100% recovery following 4 h freezing treatment.

### Cysteine blocking in cryobiosis

Tardigrades (30 animals per sample, 4 replicates per condition) were collected in microcentrifuge tubes were subjected to a 30-min incubation in 30 μM NEM or water (control). Following incubation, tardigrades were rinsed twice with 1 mL of deionized water to remove remaining blocking reagent and centrifuged at 10,000 x*g* for 3 min. Following the second rinse, the supernate was removed, and tardigrades were transferred to 35-mm Petri dishes in less than 10 μL final volume and placed in a Thermo Fisher Scientific Nalgene ‘Mr. Frosty’ Freezing Container containing 70% isopropyl alcohol at -80 ⁰C for 4 h. Tardigrades were then removed from the freezer, allowed to thaw to room temperature, and counted for survival, where survival is defined by tardigrades exhibiting controlled movement of limbs and/or body.

Imaging cryobiotic tardigrades is challenging as it is impossible to maintain below freezing conditions at the microscope. In house experimentation showed that if frozen tardigrades are put directly in chilled methanol (final concentration 12.5% v/v), their morphology did not change once the ice thawed, as confirmed from viewing under a light microscope. To image cryobiotic tardigrades, 10 μL of thawed but cold 25% (v/v) methanol/water solution previously kept at -80⁰C was added to the 10 μL frozen droplet containing bears (to reach a final methanol concentration of 12.5% v/v) within 0.12 mm deep SecureSeal^TM^ Imaging Spacers.

### Methanol blocking in osmo- and cryobiosis

Methanol can be used to stop cell signaling and arrest cellular metabolism [36–40]. In order to determine if cell signaling is required for tun formation and tardigrade survival, we exposed tardigrades to methanol before inducing cryptobioses by freezing, exposure to 600 mM sucrose, or exposure to 150 mM CaCl_2_. Tardigrades (30 per replicate, 4 replicates per condition) were incubated in 12.5% v/v methanol in a 35 mM Petri dish for 5 min before adding either 600 mM sucrose, 150 mM CaCl_2_, or freezing. Results were compared with those of non-methanol conditions to establish the reliance on cell signaling for cryptobiotic exposure. For cryobiosis, methanol exposed tardigrades were frozen in a Thermo Fisher Scientific Nalgene ‘Mr. Frosty’ Freezing Container containing 70% isopropyl alcohol at -80 ⁰C for 4 h, after which they were removed and allowed to thaw to room temperature. Tardigrades were monitored for survival and compared to tardigrades who underwent cryobiosis without methanol exposure.

### Quantification of reduced thiols

Tardigrades (1000 animals per sample, 4 samples per condition) collected in microcentrifuge tubes. There were a total of 12 samples, including controls (active tardigrades, not in cryptobiosis), osmobiosis via 75 mM CaCl_2_, osmobiosis via 600 mM sucrose, and cryobiosis, all induced as described earlier. Following cryptobiote induction for each respective condition, proteins were immediately extracted. Tardigrade proteins were extracted using a modified acid extraction [41]. Tardigrades were incubated in concentrated trifluoroacetic acid (Sigma Aldrich, St. Louis, MO) for 40 min before neutralizing to approximately pH 7 with 2 M Tris (Thermo Fisher Scientific, Waltham, MA) in water. Proteins were precipitated in 70% (v/v) ethanol (Thermo Fisher Scientific) overnight at -20°C before centrifuging at 3,220 x*g* for 1 h and collecting the resulting pellet.

For those induced into cryobiosis, frozen samples were homogenized via a plastic pestle before being subjected to protein extraction. Following precipitation, protein pellets were resuspended in 4 M urea in PBS at pH 7.5 and quantified with a CB-X assay (G-Biosciences, St. Louis, MO) against bovine serum albumin standards. Proteins were enzymatically digested with 0.5 ug Trypsin Gold (Promega, Madison, WI) for 16 h, shaking at 850 RPM at 37°C. Samples were dried in a CentriVap (Labconco, Kansas City, MO) and subsequently resuspended in PBS at pH 7.5. Resuspended samples were subjected to reduced thiol quantitation using the Measure-iT^TM^ Thiol Assay Kit (Thermo Fisher Scientific, Waltham, MA) against reduced glutathione following the manufacturer’s protocol. Briefly, the Measure-iT^TM^ thiol quantitation reagent was diluted 1:100 in PBS. In a 96-well plate, 100 uL of the diluted thiol quantitation reagent were added to 10 uL of sample/standard. Samples were incubated at room temperature for 5 min before fluorescence was measured using a SpectraMax M5 (Molecular Devices, San Jose, CA) microplate reader with excitation/emission maxima at 490/520 nm. Thiol concentrations were normalized to total protein amounts in each sample. Statistical testing was performed using a Student’s t-test.

### Confocal fluorescence microscopy

Tardigrades (either active or in tuns) were exposed to 30 μM fluorescein-5-maleimide (Sigma Aldrich, St. Louis, MO, USA) in 50 mM PBS, pH 7.2, in 12% (v/v) methanol for 2 h at room temperature. Following incubation, tardigrades were centrifuged at 10,000 x*g* for 5 min, before removing the supernate and rinsing with 1 mL of 50 mM PBS, 12% methanol (v/v), pH 7.2. Brightfield contrast and fluorescence images were acquired on a Zeiss LSM710 confocal laser-scanning microscope using a PlanApo 40X objective lens. Brightfield and fluorescence images were collected using 561-nm and 480-nm lasers, respectively, on Zeiss ZEN software (Car Zeiss, INC. NY, USA).

### Forced tun emergence via reduction

To determine if chemical reduction could induce reemergence from tuns, chemobiotic tardigrades induced into tuns by 750 μM hydrogen peroxide were dosed with concentrated (14.3 M) β-mercaptoethanol (BME or 2-mercaptoethanol, Sigma Aldrich, St. Louis, MO). Tardigrades were monitored using a dissection microscope to observe linearization, whereby tardigrade limbs returned to erect positions and bodies increased to active tardigrade size. Tardigrades were counted manually to determine total reemergence, and survival was monitored after 24 h.

### Chemical inhibition of tardigrade tuns

Tardigrades were plated one organism per well in a clear 96-well plate in 1-μL droplets of water. Tardigrades were incubated for 1 h with a working concentration of 100 μM of all inhibitors from the SCREENWELL Redox Library (Enzo Life Sciences, Farmingdale, NY) and monitored for physiological changes. There were approximately 10 tardigrades monitored per inhibitor in the SCREENWELL library. Following this incubation, tardigrades were incubated in a working concentration of 75 mM CaCl_2_ and observed for tun formation after 3 h.

Glutathione (reduced and oxidized), L-ergothioniene, and B-lapachone were studied using 30 tardigrades per sample in 4 replicates per inhibitor. Each was applied with a working concentration of 75 mM and 0.1% DMSO to improve permeation. Following incubation for 1 h, tardigrades were treated in 75 mM CaCl_2_ and observed for tun formation after 3 h.

### Electron paramagnetic resonance spectroscopy

Aliquots of the superoxide-reactive spin trap 1-hydroxy-3-methoxycarbonyl-2,2,5,5-tetramethylpyrrolidine (CMH, 1 mM final concentration) (Enzo Life Sciences, Farmingdale, NY) were prepared in modified Krebs-HEPES buffer containing 99.01 mM NaCl, 4.69 mM KCl, 2.50 mM CaCl_2_·2H_2_O, 1.20 mM MgSO_4_·7H_2_O, 29.76 mM NaHCO_3_, 1.04 mM KH_2_PO_4_, 11.10 mM D+ glucose, 20.00 mM Na^+^-HEPES, 25 μM deferoxamine mesylate, and 5 μM diethyldithiocarboxamic acid ammonium salt (all reagents from either Thermo Fisher Scientific or Sigma Aldrich). CMH aliquots were stored at -20°C until use. For each of 7 trials (stressed, n = 3; non-stressed, n = 4), a sample of 200 *H*. *exemplaris* tardigrades with minimal algae was collected and centrifuged at 13,400 x*g*. The resultant supernate was removed until the final volume of culture sample remaining was 10 μL. For each trial, timing was begun upon transfer of an aliquot of CMH from storage to ice. Prior to adding CMH to samples, the CMH was held under nitrogen gas flow for five min to remove dissolved oxygen. Measurements were collected on an X-band EMXPlus Spectrometer (Bruker, Billerica, MA) with the following parameters: sweep width, 100 G; center field, 3510 G; modulation amplitude, 1.000 G; receiver gain, 30; microwave power, 10.02 mW; number of scans, 4. Across trials, samples were time-matched with respect to transferring the CMH to ice (e.g., all samples received CMH at the same time post transfer, were placed into the instrument at the same time post transfer, and data was collected at the same time post transfer). Stressor solution or deionized water and CMH (1:1, v/v; 500 μM working concentration) were added 2 min prior to the collection of data for the initial timepoint. Samples were wicked into 50 μL capillary tubes (Sigma Aldrich, St. Louis, MO). Data points were collected at 5 min intervals (beginning at 2 min post-addition of CMH/stressor) for 30 min. Signal intensity for all data points was determined using the difference between the maximum intensity and minimum intensity of the CMH signal and then normalized to the number of animals. The rates of CMH oxidation between timepoints were determined using the rate of signal change between points on a signal intensity per-animal versus time plot.

### Chemical inhibition with erastin

To determine the role of VDAC2 on tardigrade tun formation, erastin inhibition was conducted using 3 replicates of 30 tardigrades, each tardigrade in an individual well of a 96-well plate. Tardigrades were incubated in a 100 nM working concentration of erastin (MedChemExpress, Monmouth Junction, NJ) in 0.1% DMSO overnight. After 20 h, tardigrades were observed under a dissecting light microscope and manually counted to determine the number of tuns formed.

## Results and discussion

### ROS exposure induces tun formation in tardigrades–direct evidence of chemobiosis

**Fig 1:**
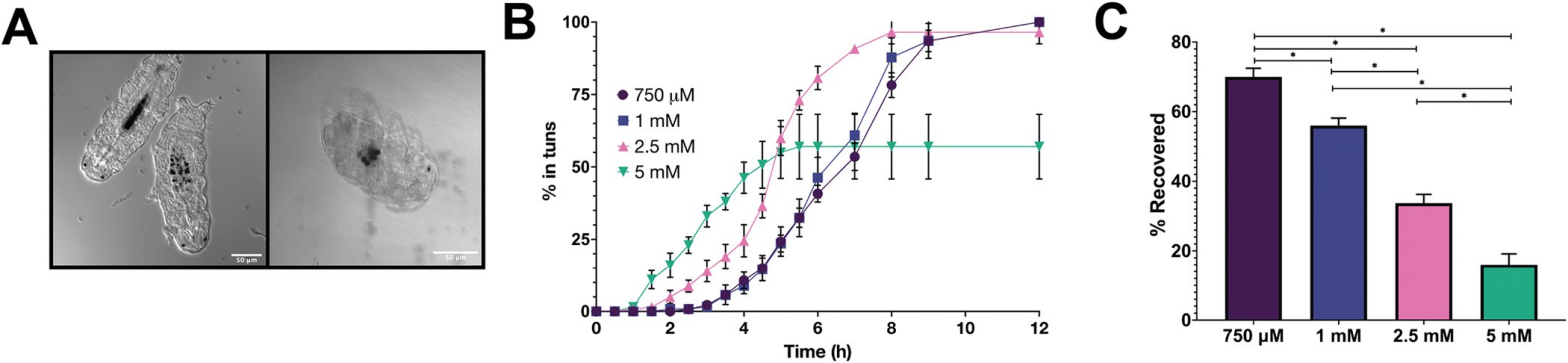
Peroxide induces tuns in a concentration dependent manner in *Hypsibius exemplaris*. A) Confocal images of tardigrades in the hydrated state as well as a H_2_O_2_– induced tun. Tuns are about 25% of the size of the hydrated tardigrades. B) The percent of tardigrades entering tuns following different concentrations of H_2_O_2_. Tardigrades were measured in three biological replicates, where a single replicate included 25–30 tardigrades. Error bars represent standard deviation. C) The percentage of tardigrades that reemerged from H_2_O_2_– induced tuns following reintroduction to distilled water. Error bars represent standard deviation. Only tardigrades that enter tuns are counted toward total survival. Statistical significance was determined by paired t-tests with Welch’s correction, where * indicates p < 0.05.

Tardigrades survive multiple stressors that are prone to generating ROS *in vivo*, including desiccation, extreme fluctuation in osmolarity, and freezing, among others [14, 42–45]. We therefore hypothesized that ROS may play a crucial role in signaling tun induction in response to environmental fluctuations. This was explored using exogenously applied H_2_O_2_ and monitoring the physiological effect(s) on tardigrade morphology (**Fig 1**). Tardigrades exhibited a dose-dependent response to exogenous H_2_O_2_, with higher concentrations generating tuns within an hour of exposure. Tardigrades exposed to peroxide retracted their limbs and decreased their size to approximately 25% of their hydrated counterparts, thus indicating chemobiosis resulting in tun formation had taken place (**Fig 1A**). To further explore this phenomenon, tardigrade tun formation was monitored over time for varying concentrations. Measurements were conducted in 3 biological replicates, with a single replicate including 25–30 specimens. Time to tun formation was distinct across peroxide concentrations, with 5 mM H_2_O_2_ resulting in tuns for 10% of the tardigrades by 90 min of exposure, while 750 μM H_2_O_2_ took at least 4 h to achieve the same result (**Fig 1B**). All tardigrades entered tuns following exposure to 750 μM H_2_O_2_ by 12 h, whereas it took only 8 h with 2.5 mM H_2_O_2_. Only 57% of tardigrades exposed to 5 mM H_2_O_2_ entered tuns; the remaining 43% entered a turgid, semi-translucent state indicative of tardigrade death. The lethality of higher ROS concentrations could indicate a risk of over-oxidation (perhaps in the form of irreversible oxidation, in contrast to reversible) in extreme conditions. This was further mirrored by a significantly decreased tardigrade viability at all concentrations >1 mM H_2_O_2_, with only 34% survival following exposure to 2.5 mM H_2_O_2_ and 16% following exposure to 5 mM H_2_O_2_, both determined by recovery of motility following emergence from tuns (**Fig 1C**). In contrast, concentrations of 1 mM and 750 μM H_2_O_2_ resulted in post-tun tardigrade viability of 57.4 and 70.0%, respectively. This is markedly lower than the survival of *Echiniscus blumi* exposed to H_2_O_2_, unsurprising due to the decreased stress survival of *H*. *exemplaris* frequently reported in literature [46, 47].

Despite the absence of desiccation or significant fluctuations in osmolarity, tardigrades still expelled internal water stores and initiated tun formation, resulting in an approximate 75% decrease in body length (**Fig 1A**). This suggests that water expulsion during tun formation is an active process rather than the result of spontaneous flux; this corroborates previous work in the tardigrade species *Richtersius coronifer* that reported an 87% volume reduction following anhydrobiosis that was dependent on mitochondrial activity [23]. This previously reported dependence on mitochondrial activity combined with the results reported herein suggests that the water expulsion may be mediated by oxidation or oxidative signaling. This process was quickly reversed when tuns were placed back in distilled water in the absence of exogenous ROS, suggesting that tardigrades are able to rapidly sense environmental changes to initiate anatomical transitions. Tardigrades’ ability to undergo chemobiosis has previously been predicted, but the data presented here is the first empirical evidence of chemical-induced tun induction.

### *H*. *exemplaris* can be reproducibly induced into cryo- and osmobiosis

**Fig 2:**
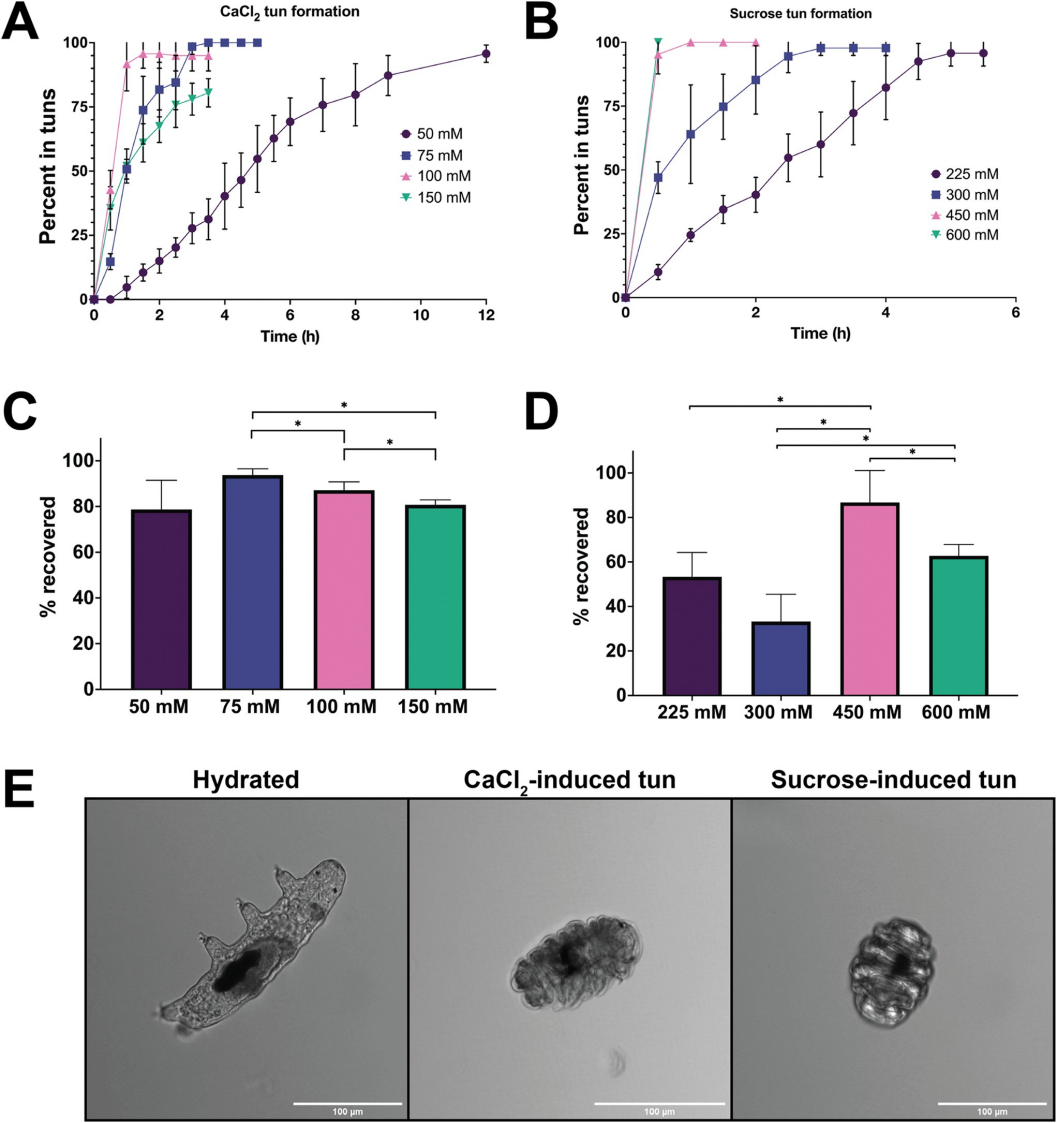
Tardigrades can be induced via osmobiosis using both CaCl_2_ and sucrose. A) The percent of tardigrades entering tuns following different concentrations of CaCl_2_. Tardigrades were measured in three biological replicates, where a single replicate included 25–30 tardigrades. B) The percent of tardigrades entering tuns following different concentrations of sucrose. Tardigrades were measured in three biological replicates, where a single replicate included 25–30 tardigrades. C) The percentage of tardigrades that reemerged from CaCl_2_–induced tuns following reintroduction to aqueous media. D) The percentage of tardigrades that reemerged from sucrose–induced tuns following reintroduction to aqueous media. A-D) Error bars represent standard deviation. C-D) Statistical significance was determined by paired t-tests with Welch’s correction, where * indicates p < 0.05. E) Tardigrades imaged using a confocal microscope in their hydrated, CaCl_2_-induced, and sucrose-induced states.

While cryobiosis has been previously explored in *H*. *exemplaris*, osmobiosis required thorough empirical characterization. We mapped the induction and concentration of osmobiosis using two distinct osmolytes, CaCl_2_ and sucrose, chosen due to their ionic and non-ionic natures (**Fig 2**). Both stressors were applied in concentrations far exceeding expected physiological concentrations, as it was essential to induce a stress response that would prove fatal should the tardigrades not enter their resilient tun form. As observed with peroxide, tun inductions by CaCl_2_ and sucrose were highly concentration dependent. While 50 mM CaCl_2_ resulted in total tun formation in 12 h, 75 mM CaCl_2_ produced the same result in only 3.5 h (**Fig 2A**). Exposure to 100 and 150 mM CaCl_2_ resulted in 92% and 52% of tuns induced after 1 h, respectively; interestingly, these higher concentrations also resulted in tardigrade fatality before tun formation could occur. Survival was high in all concentrations for those that induced tuns following CaCl_2_ exposure, with an average survival rate of 80–90% for all concentrations (**Fig 2C**). Sucrose also formed tuns in a concentration-dependent manner (**Fig 2B**); however, unlike peroxide and CaCl_2_ exposure, higher concentrations did not result in tardigrade death prior to tun formation. This is consistent with osmobiosis mapped in the tardigrade species *Ramazzottius oberhaeuseri*, which had a higher tolerance to nonionic osmolytes in comparison to ionic [45]. All tardigrades exposed to 225 mM, 300 mM, 450 mM, and 600 mM sucrose formed tuns, with 100% of the tardigrades forming tuns at 5.5, 4, 2, and 0.5 h, respectively. Interestingly, the survival rate post-tun formation was not concentration dependent, with repeated trials producing the same pattern of survival that is inconsistent with dose concentration (**Fig 2D**). While deviation in tardigrade size and/or age could generate stochasticity in the data, these differences would be expected to be random. The repeated findings of non-correlative survival rates suggest that this is a regulated phenomenon rather than random. Further investigation is needed before a conclusion can be made.

Both CaCl_2_ and sucrose can reproducibly produce tuns with high rates of post-stressor survival (**Fig 2**). Physiologically, there appears to be slight differences between CaCl_2_ and sucrose-induced tuns, with sucrose tuns appearing more compact (**Fig 2E**). This was reflected in volume estimations, where sucrose induced tuns appear to have a slightly smaller overall volume than CaCl_2_ induced (**S1 Fig**). This could be due to the higher calculated osmotic pressure (π) in sucrose, with 15.17 bar compared to 9.59 bar; however, more investigation is needed to determine the precise cause. Interestingly, this tun formation and survival was not consistent across all osmolytes. Glucose and glycerol exhibited nearly total tun formation at 600 mM, but survival was less than 50%; in contrast, neither NaCl or MnCl_2_ formed reproducible tuns in repeated trials. This suggests that osmotic pressure alone is not the inducer of tun formation, and that intracellular regulatory mechanisms must have some control over tun initiation.

### Reversible cysteine oxidation is essential for tun formation

**Fig 3:**
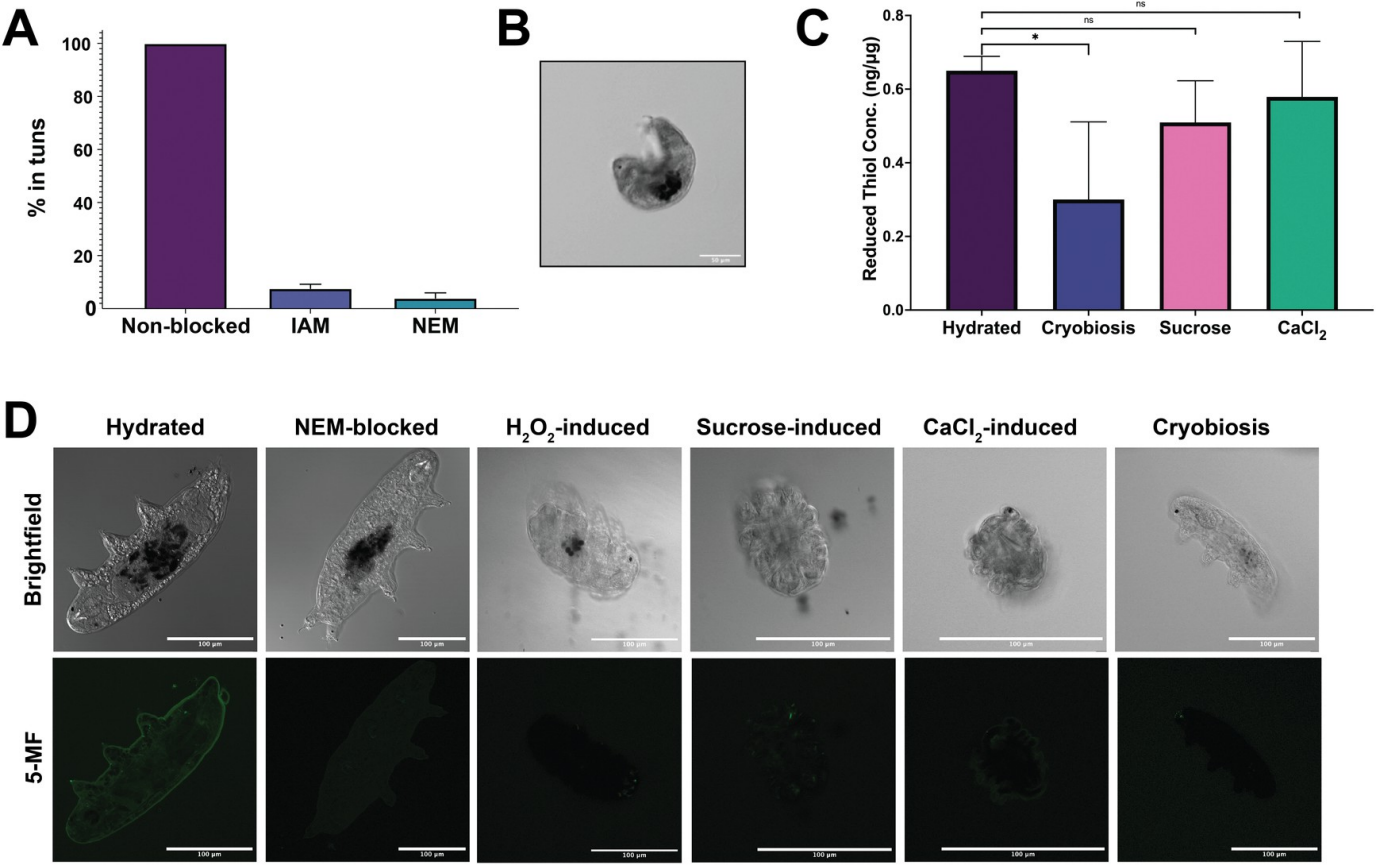
Blocking of cysteine residues prevents tun formation. A) Tardigrades exposed to either IAM or NEM, both of which bind irreversibly to reduced cysteines, significantly decreased tun formation in H_2_O_2_–exposed tardigrades. B) Confocal image of a NEM-blocked tardigrade following exposure to H_2_O_2_. C) Tardigrade proteins were extracted and digested with trypsin before fluorescent quantification of reduced cysteine thiols. Thiol concentration was normalized to protein concentration. A, C) Error bars represent standard deviation. Statistical significance was determined by paired t-tests with Welch’s correction, where * indicates p < 0.05. D)Tardigrades were labeled with 5-MF before imaging with a confocal microscope. To demonstrate the selectivity of the label, one set of tardigrades was blocked with NEM prior to 5-MF labeling, showing significantly diminished fluorescence compared to the unblocked tardigrade.

ROS contribute to cellular homeostasis through rapid reversible oxidative signaling through which the oxidation of cysteine thiols is used to trigger protein responses [48]. Therefore, ROS induction of tun formation through the oxidation of cysteine thiols was examined by blocking reduced cysteine thiols in hydrated tardigrades with either iodoacetamide (IAM) or *N*-ethylmaleimide (NEM), both of which irreversibly bind to reduced cysteine thiols and block oxidation [49]. Tardigrades were incubated in either 30 μM IAM or NEM (concentration chosen following titration series to ensure tardigrade survival) and exposed to 750 μM H_2_O_2_ for 12 h (**Fig 3A**). Experiment was conducted with 3 biological replicates, where each biological replicate included 30 specimens. While 100% of tardigrades formed tuns in the absence of Cys-blocking, only 7.6 and 4.0% of tardigrades blocked with IAM and NEM, respectively, formed tuns by 12 h. This dramatic decrease in tun formation indicates an essential role for cysteine oxidation in the mechanism and/or signaling of the process. While a small difference was observed in IAM compared to NEM, this difference was not statistically significant, and likely represents the slightly higher binding affinity and specificity of NEM compared to IAM [50, 51]. Tardigrades exposed to NEM before being exposed to peroxide showed decreased size compared to fully hydrated, and visible deformity with limbs extended rather than retracted (**Fig 3B**), suggesting that cysteine oxidation is necessary for limb retraction during tun formation.

Whereas chemo- and osmobiosis form tuns, cryobiosis does not; it was therefore unclear whether or not oxidation would still be implicated in cryobiosis. To assess this phenomenon, cryobiosis was analyzed to ensure high survival rates, with a freezing rate of 1°C/min resulting in 87% survival (**S2A Fig**). Cryobiotes imaged using a confocal microscope revealed a distinct morphology from osmo- or chemobiotes, with the tardigrades appearing more globular rather than the ordered, compact form of tuns (**S2B Fig**). To determine if cysteine oxidation was necessary for survival, tardigrades were exposed to 30 uM NEM prior to freezing. This resulted in tardigrades becoming more linear and distended during freezing, likely contributing to the lack of any survivors following reheating to room temperature (**S2C Fig**). Based on this data, it appears that cysteine oxidation is still necessary for cryptobiotic survival, even when the tun formation is not involved.

In addition to blocking reversible oxidation of cysteine thiols using NEM or IAM, cryptobiosis can be blocked using 12.5% (v/v) methanol. Methanol quenching is a well-established method to stop cell signaling events and arrest metabolism [36–40]. Previous work has used methanol to reversibly and non-lethally anesthetize *C*. *elegans;* [34] we therefore titrated methanol into tardigrades and determined that 12.5% (v/v) resulted in complete cessation of movement and 100% survival following methanol removal. Following methanol exposure, tun formation was inhibited by 91% and 99% in sucrose- and CaCl_2_-exposed samples, respectively, and cryobiote formation was inhibited by 100% (**Table 1**). If tun generation was the result of desiccation via passive osmosis, we would expect methanol-dosed tardigrades to still generate tuns following application of sucrose, CaCl_2_, and/or cryobiotic conditions. Thus, the blocking of tun formation by methanol gives further evidence that tun initiation is not the result of desiccation via passive osmosis; rather, it is a highly regulated phenomenon, likely mediated by signaling mechanisms that can be quenched with methanol.

**Table 1:** Percentage of tardigrades that undergo either osmobiosis or cryobiosis following exposure to methanol. Biological replicates included 30 specimens each and 4 replicates. The sucrose and CaCl2 conditions were monitored for tun formation, whereas the cryobiotes were monitored for survival following freezing. None of the cryobiotic tardigrades survived.

	*+ Methanol*		*- Methanol*	
Condition	Mean	SD	Mean	SD
Sucrose	9.01	2.98	100.0	0
CaCl_2_	0.47	0.94	100.0	0
Cryobiosis	0.00	0.00	87.48	5.11

For quantitative assessment of thiol oxidation, reduced thiols were quantified from the tardigrade proteome using a fluorescence-based quantitative thiol assay. Proteome digestion was essential to increase accessibility to internal cysteines that may be sterically hindered from binding to the fluorophore. To circumvent variation due to tardigrade size and/or age, quantified cysteine thiols were normalized to quantified protein content. All cryptobioses exhibited lower concentrations of reduced cysteines (**Fig 3C**). Cryobiosis had the lowest of all stressors, with approximately half of the free cysteines compared to the control (**Fig 3C**). This suggests differential cysteine regulation across stressors.

Further qualitative assessment of cysteine thiol oxidation was conducted using confocal microscopy with fluorescein-5-maleimide (5-MF), a labeling agent that irreversibly derivatizes cysteine thiols, across all inducible stressors (**Fig 3D**). Active tardigrades exhibited fluorescence, with 5-MF localized near the tardigrade epidermis, whereas tuns and cryobiotes exhibited minimal fluorescence. This indicates that reduced cysteine thiols in the active, hydrated tardigrades were likely oxidized and inaccessible to 5-MF labeling once in the tun state. As with the thiol quantification assay, cryobiosis exhibited the least amount of fluorescence with 5-MF. Interestingly, the remaining cryptobiotes also have diminished fluorescence compared to the control, with much less of the body showing any fluorescent signal.

Cysteines can rapidly undergo a variety of reversible oxidative modifications, including sulphenylation, disulfide bond formation, glutathionylation, and *S-*nitrosylation, that often facilitate rapid intracellular signaling [52, 53]. To determine the reversibility of tun-inducing cysteine oxidation, tuns induced by 12-h exposure to 750 μM H_2_O_2_ were exposed to the reductant β-mercaptoethanol (BME). After 30 min of concentrated (14.3 M) BME exposure, 98% of tardigrades had reemerged with their bodies lengthened and all 8 legs clearly visible, with emergence visualized via microscopy beginning at 5 min. Interestingly, reductant-induced tun release was lethal to all tardigrades, indicating that native tun release must be a highly regulated physiological event. This could potentially occur through crosstalk with other post-translational modifications; the rapid recovery of H_2_O_2_-induced tuns following placement in distilled water (<1 h) likely precludes an upregulation in protein expression as a contributing factor. The reversibility of tuns using BME suggests that the cysteine modification required for tun formation is reversible oxidation that can be controlled through exogenous ROS and reducing agents.

### Tardigrades may use an intricate network of ROS signals to initiate and survive tun formation

Our results indicate that the reversible oxidation of cysteine thiols is essential for tun induction in *H*. *exemplaris*. The dose-dependence of tun formation in the presence of H_2_O_2_ suggests that either environmental ROS or the intracellular production of ROS in the presence of external stressors triggers tardigrades to enter cryptobiosis. To further assess intracellular ROS generation in tun formation, tardigrades were screened using the SCREEN-WELL REDOX library, through which they were exposed to either antioxidants or oxidants, monitored for physiological changes, and then induced into tuns using CaCl_2_. Compounds that did not have a physiological effect alone but prevented tun formation following stressor introduction were examined further. In total, 84 compounds were screened, including many phenolic antioxidants, radical scavengers, and thiol-containing reducing agents. Following application of CaCl_2_, 72 of the compounds inhibited tun formation, with 15 inhibiting over 50% of tardigrades (**S1 Table**). Three of the highest inhibiting compounds in this preliminary screen were selected for re-analysis across replicates: glutathione, L-ergothioneine, and β -lapachone.

**Fig 4:**
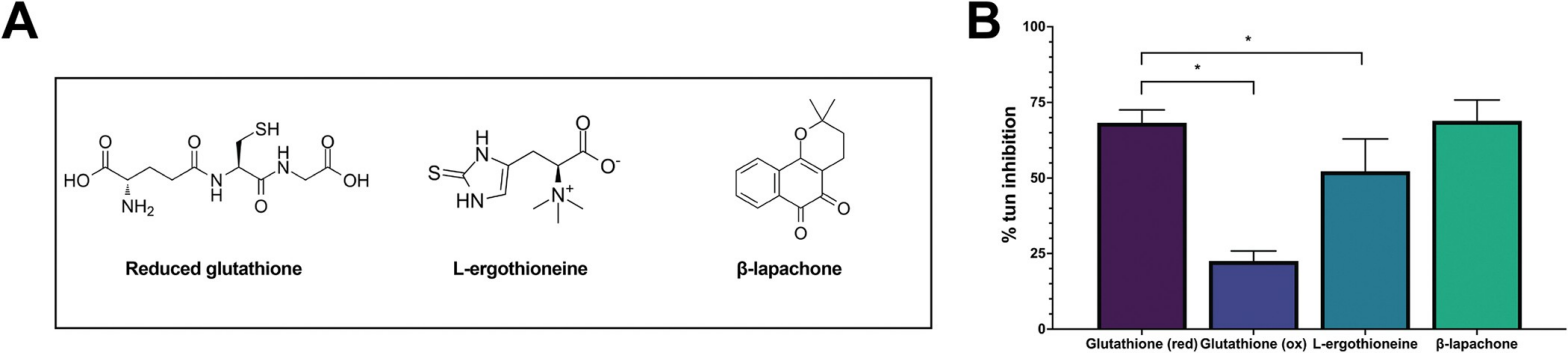
Exposure to exogenous antioxidants prior to osmotic stress decreases tardigrade survival. A) Structures of antioxidants. B) Three inhibitors were further characterized in replicates following the initial screening. Glutathione, L-ergothioneine, and B-lapachone all inhibited >50% of tuns from forming. Error bars indicate standard deviation. Statistical significance was determined by paired t-tests with Welch’s correction, where * indicates p < 0.05.

Glutathione was one of the largest inhibitors of tun formation, preventing tuns in ~68% of the tardigrades following exposure to CaCl_2_ (**Fig 4**). When oxidized glutathione was used instead, tun inhibition decreased to 22%, thus indicating that reduced glutathione has a significant effect on tun formation. This likely derives from the readily oxidized cysteine in glutathione where an influx of glutathione renders ROS unable to reach their target(s). Two other antioxidants were evaluated for their ability to prevent tun formation: L-ergothioneine, a sulfur containing small molecule, and β -lapachone, a sulfur-free benzochromenone, both of which significantly inhibited tun formation, with 52% and 69% inhibition, respectively (**Fig 4B**). The ability to suppress tun formation with antioxidants provides evidence that intracellularly generated ROS mediate tardigrade survival in the presence of exogenous stressors.

**Fig 5:**
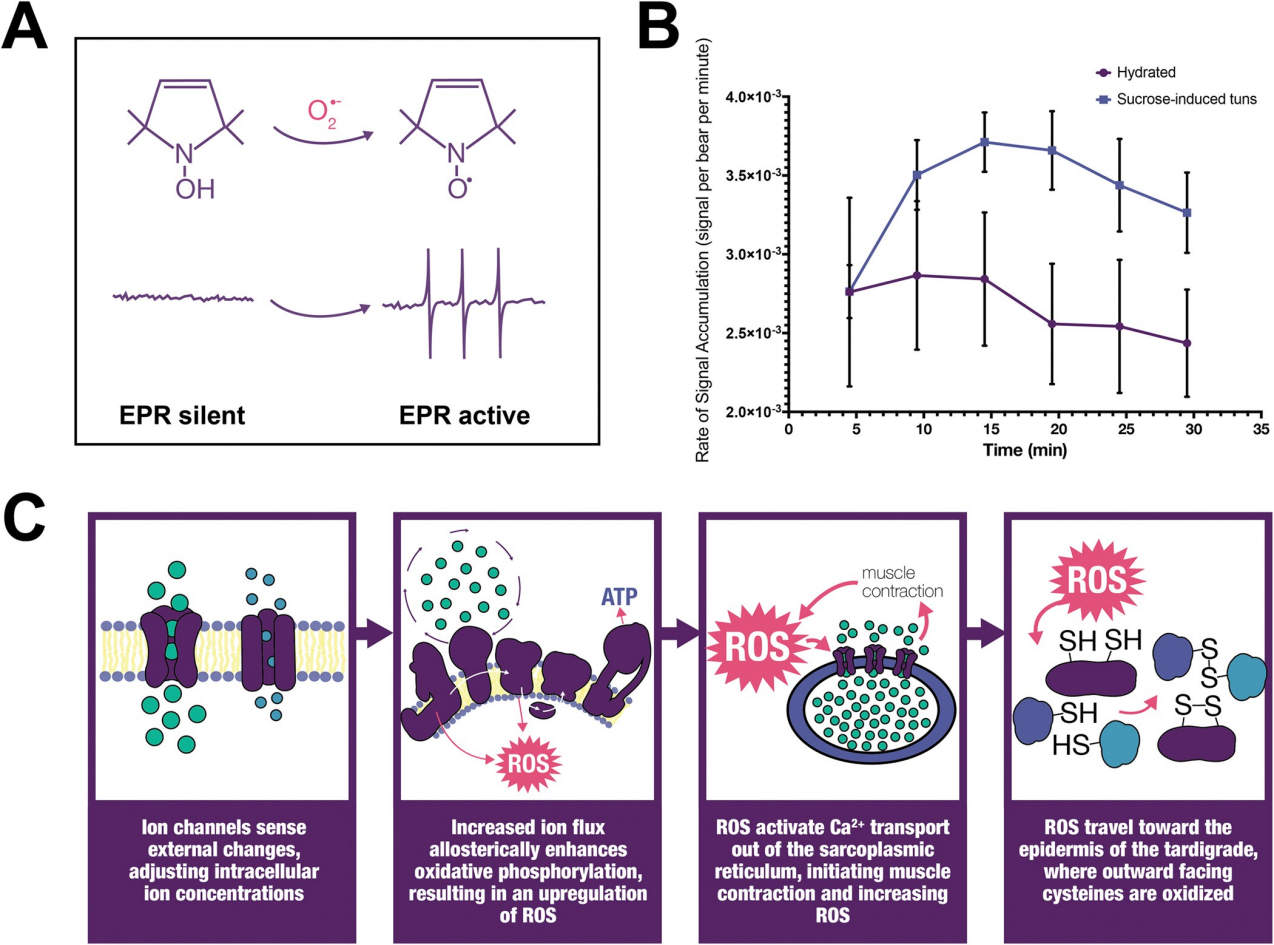
Tardigrades accumulate ROS following stress exposure. A) CMH forms a stable nitroxide radical following contact with superoxide that is quantifiable via EPR. B) Hydrated and sucrose exposed tardigrades were probed with CMH and measured with EPR every 5 min. Sucrose exposed tardigrades accumulate superoxide at significantly higher rates than non-stressed tardigrades, beginning at 10 min post stress introduction. Error bars indicate standard deviation. Statistical significance was determined by paired t-tests at each time point with Welch’s correction, where * indicates p < 0.05. C) The working hypothesis for tardigrade survival via ROS-mediated protein signaling.

EPR spectroscopy was implemented to further delineate ROS accumulation following exposure to stress. EPR has previously been implemented to successfully measure the superoxide anion radical in the tardigrade *Paramacrobiotus richtersi* [54]. Hydrated tardigrades and those exposed to 600 mM sucrose were analyzed using the superoxide-sensitive spin probe CMH [55]. Superoxide is generated by electron leak at Complex I and Complex III of the mitochondria, the rate of which is increased under stress [56, 57]. CMH is a hydroxylamine probe that generates a stable nitroxide radical that can be detected via EPR (**Fig 5A**). Exposing CMH to hydrated and sucrose-exposed tardigrades thus enabled relative quantification of superoxide production over time, allowing us to compare the rate of accumulation as tardigrades entered their osmobiotic tun. Both hydrated and sucrose-exposed tardigrades started with overlapping rate of superoxide accumulation (**Fig 5B**). By 10 min, however, sucrose significantly increased in accumulation rate compared to the hydrated sample, with an approximately 25% increase in superoxide rate. This increase continued for the duration of the 30-min measurements, thus showing that the induction of osmobiosis releases intracellular ROS.

Previous work has shown upregulation of antioxidant enzymes in the tardigrade species *Paramacrobiotus richtersi*, *Paramacroiotus spatialis*, and *H*. *exemplaris* following cryptobiosis [26, 31, 58]. With chemical treatment mirroring the effects of anhydrobiosis, it was observed that glutathione increases in *H*. *exemplaris* [26]. However, in *P*. *spatialis*, increases in ROS were observed in tardigrade storage cells with increases correlating to the amount of time in anhydrobiosis. Herein, we have observed increased rates of ROS production following stressor induction as well as the ability to block tuns from forming when induced in the presence of antioxidants above the physiological levels reported in the literature [26]. This suggests that there may be two phases of ROS accumulation: a “burst” that occurs upon stressor exposure that triggers the reversible oxidation of cysteine and leads to cryptobiosis, and a long-term accumulation that occurs following cryptobiosis induction that may maintain cryptobiosis [58]. This is further supported by the hydrogen peroxide data shown here, indicating that the removal of tun-inducing ROS results in tun emergence with high survival rates. The ability to block and/or perturb these functions with exogenous antioxidants as well as high concentrations of ROS show that this is likely a highly regulated process worthy of further investigation.

We hypothesize that tardigrades sense external changes and stressors via the activation or inhibition of ion channels (**Fig 5C**). Mitochondrial ROS are modulated by ion fluxes (particularly calcium), which allosterically increase mitochondrial electron transport via complex II (**Fig 5C**). We therefore hypothesized that a critical regulatory protein in tardigrade survival would be voltage-gated anion channel 2 (VDAC2), a highly abundant mitochondrial channel responsible for the majority of calcium flux in and out of the mitochondria. To determine the influence of VDAC2 on tun formation and tardigrade survival, tardigrades were exposed to the VDAC2 inhibitor erastin and monitored for physiological changes. Following exposure to 100 nM of erastin, tardigrades began to form tuns within minutes. Within 24 h, 75.56% (SD: 6.94) of the tardigrades had formed tuns. This suggests that the flux of calcium in and out of the mitochondria is likely implicated in tun formation and that VDAC2 closes to decrease flux in response to external stressors.

## Conclusion

Tardigrades have long been renowned for their remarkable ability to survive adverse conditions, an achievement that has helped them to survive fluctuating environments since they evolved in the Cambrian era. However, the mechanisms used to sense and respond to external stressors in order to initiate survival strategies have not previously been described. We have revealed that tardigrade survival is dependent on reversibly oxidized cysteines coordinating the entrance and emergence from survival states in a highly regulated manner. Through implementation of EPR and redox library screens, we have demonstrated that intracellular release of ROS is essential for tun formation. We have also characterized the initiation, emergence from, and dose-dependence of both osmobiosis and chemobiosis, shown here in *H*. *exemplaris* for the first time. The rapid induction of tardigrade tuns via osmobiosis, paired with the high survival rates following emergence, makes it an easily implemented cryptobiotic state for further exploration of the mechanisms of tardigrade survival.

## Supporting information

S1 Fig Tardigrade volume estimation when hydrated (control), induced into tuns with 75 mM CaCl_2_, and induced into tuns with 600 mM sucrose. (TIF)

S2 Fig Tardigrades rely on cysteine oxidation to survive cryobiosis. **A)** The survival of tardigrades that underwent cryobiosis for 24 h. Survival was determined by coordinated leg movements following return to room temperature. **B)** Confocal image of a tardigrade undergoing cryobiosis. Note that cryobiosis does not facilitate tun induction. **C)** Confocal image of a tardigrade exposed to 30 uM NEM prior to undergoing cryobiosis. None of the NEM-blocked tardigrades survived cryobiosis.
(TIF)

S1 Table Results of tardigrade inhibition study. Tardigrades were screened with the SCREENWELL Redox Library (Enzo Life Sciences) to determine detrimental effects to tun formation. Tardigrades were exposed to 100 uM (working concentration) of each compound and monitored for fatalities after 1 h. Following the hour, tardigrades were exposed to 75 mM CaCl_2_ for 3 h, before monitoring for tun formation. Percentage of inhibition refers to the number of tardigrades (that were not deceased after 1 h) not in tuns following 3 h of CaCl_2_.
(XLSX)
