# Supplementary figures and images for: Chemobiosis reveals tardigrade tun formation is dependent on reversible cysteine oxidation
Source: PLoS One. 2024 Jan 17;19(1):e0295062. doi: 10.1371/journal.pone.0295062 (PMC10793892; doi:10.1371/journal.pone.0295062)

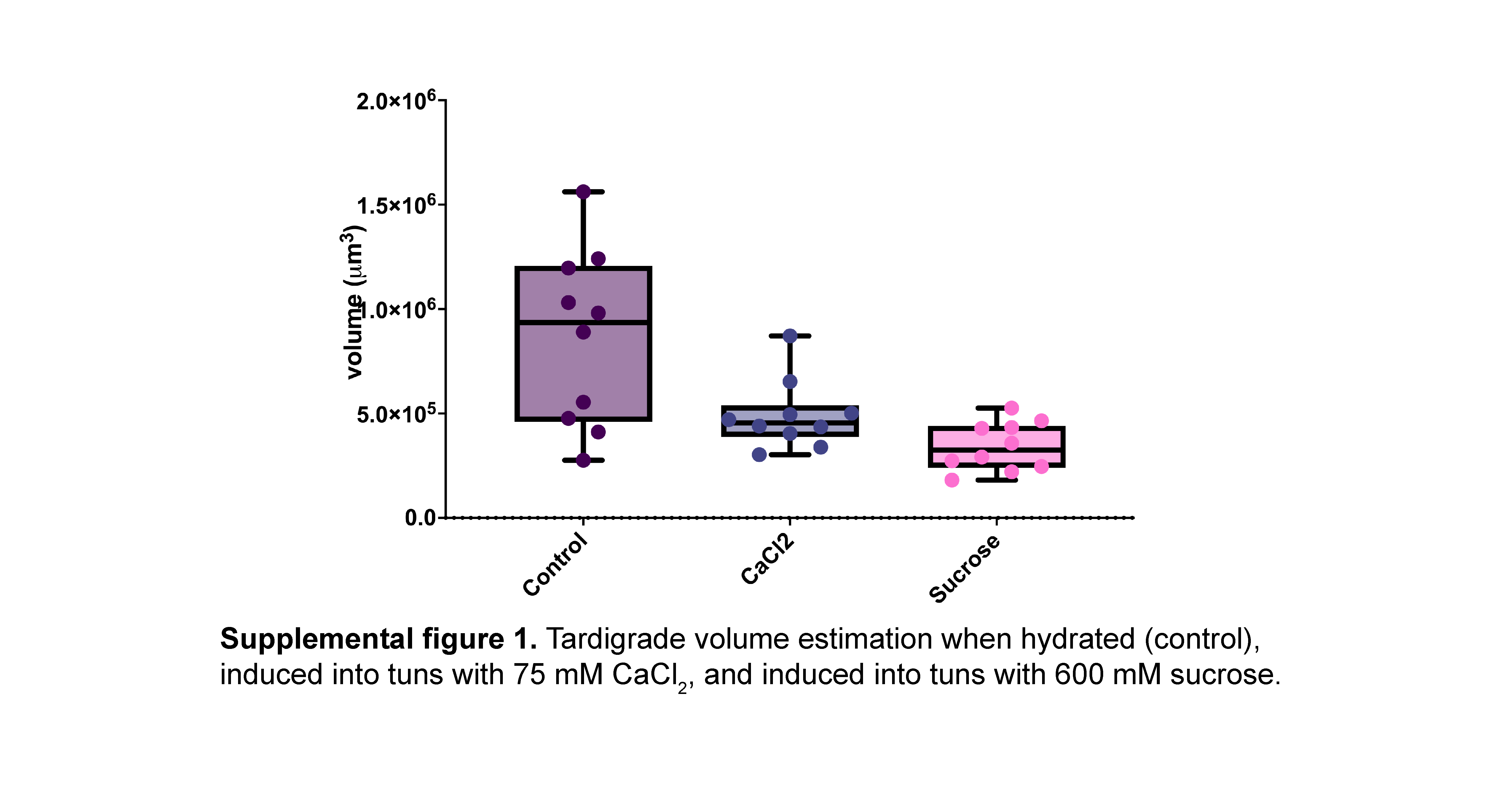

Supplement: S1 Fig — (TIF) [file pone.0295062.s001.tif]

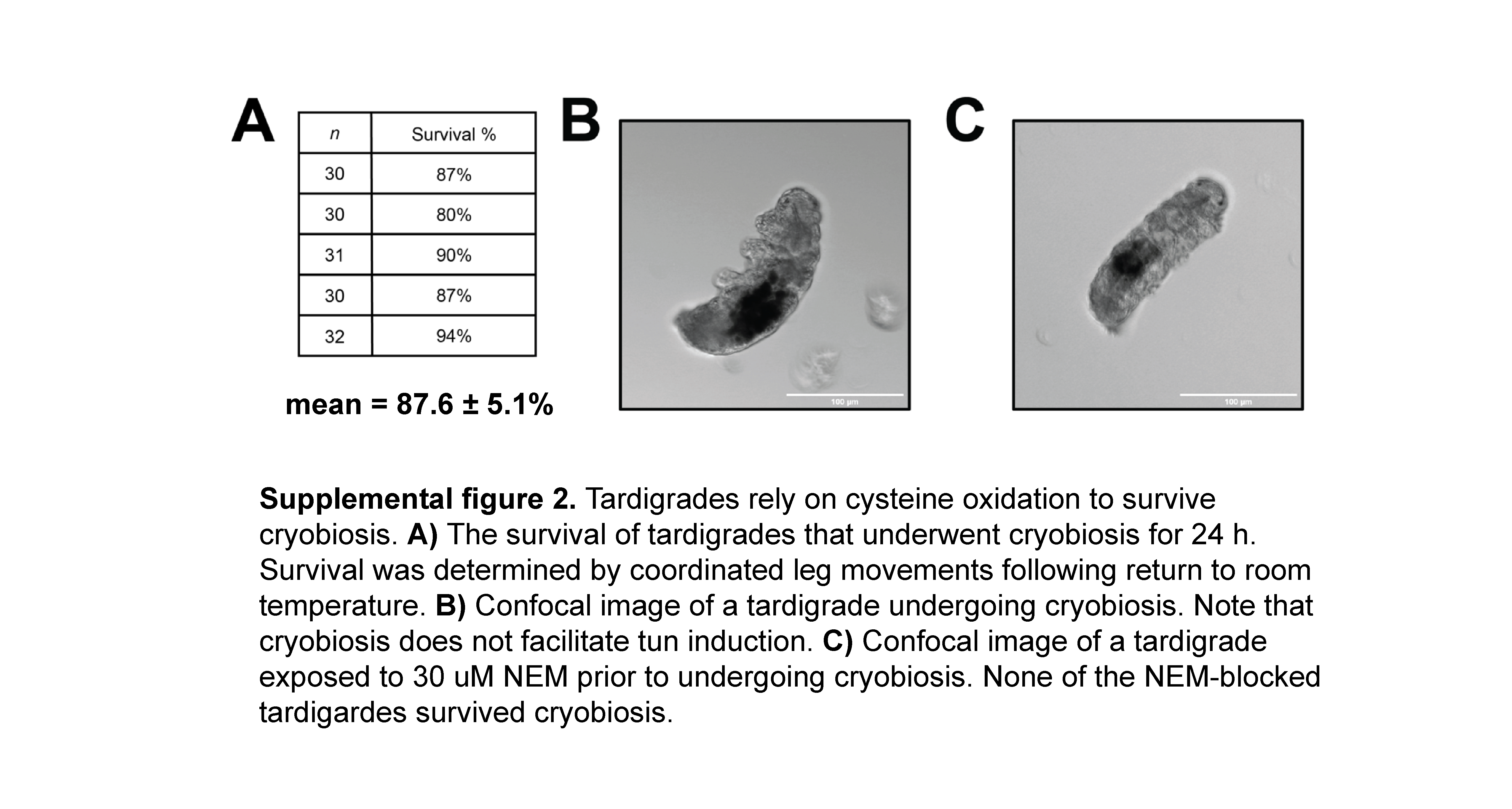

Supplement: S2 Fig — A) The survival of tardigrades that underwent cryobiosis for 24 h. Survival was determined by coordinated leg movements following return to room temperature. B) Confocal image of a tardigrade undergoing cryobiosis. Note that cryobiosis does not facilitate tun induction. C) Confocal image of a tardigrade exposed to 30 uM NEM prior to undergoing cryobiosis. None of the NEM-blocked tardigrades survived cryobiosis. (TIF) [file pone.0295062.s002.tif]
